# Iron Oxychalcogenides and Their Photocurrent Responses

**DOI:** 10.1021/acs.inorgchem.3c03672

**Published:** 2024-02-02

**Authors:** Sandy Al Bacha, Sébastien Saitzek, Houria Kabbour, Emma E. McCabe

**Affiliations:** † Univ. Lille, CNRS, Centrale Lille, ENSCL, Univ. Artois, UMR 8181–UCCS–Unité de Catalyse et Chimie du Solide, F-59000 Lille, France; ‡ University of Kent, School of Physical Sciences, Canterbury, Kent CT2 7NH, U.K.; § Department of Physics, Durham University, Durham DH1 3LE, U.K.; ∥ Univ. Artois, CNRS, Centrale Lille, Univ. Lille, UMR 8181, Unité de Catalyse et Chimie du Solide (UCCS), F-62300 Lens, France

## Abstract

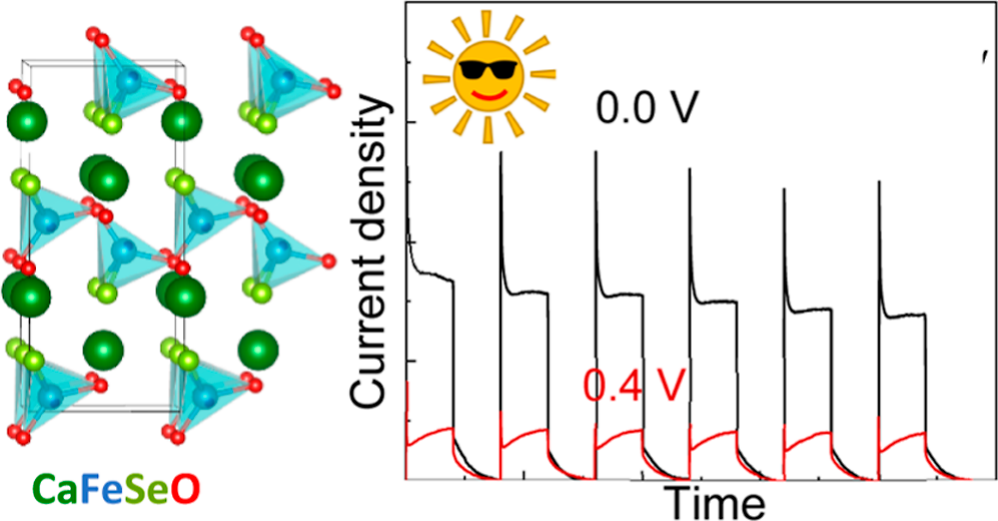

We report here the results of an experimental investigation of the electronic properties and photocurrent responses of the CaFeO*Q* and La_2_O_2_Fe_2_O*Q*_2_ phases and a computational study of the electronic structure of polar CaFeOSe. We find that both CaFeO*Q* (*Q* = S and Se) have band gaps and conduction band edge positions compatible with light-driven photocatalytic water splitting, although the oxysulfide suffers from degradation due to the oxidation of Fe^2+^ sites. The higher O/*Q* ratio in the Fe^2+^ coordination environment in CaFeOSe increases its stability without increasing the band gap beyond the visible range. The photocurrent CaFeOSe shows fast electron–hole separation, consistent with calculated carrier effective masses. These results suggest that these iron oxychalcogenides warrant further study to optimize their stability and morphology for photocatalytic and other photoactive applications.

## Abstract 2

Iron oxychalcogenides are investigated for photoactivity. Our experimental work (including photocurrent and photocatalysis measurements) is complemented by DFT studies to demonstrate the potential of iron oxychalcogenides for photoactive behavior. In particular, the polar oxyselenide CaFeSeO is experimentally shown to have fast electron−hole separation and migration, consistent with the low values calculated for electron and hole effective masses.

## 1 Introduction

Water splitting photocatalysis reactions have the potential to generate hydrogen in a clean and sustainable way if they can be carried out under solar irradiation. However, this imposes constraints on the magnitude of the photocatalyst’s band gap of 1.23–3.00 eV and the band edge positions [conduction band minimum (CBM) is more negative band than the reduction potential of H_2_O/H_2_ (0 V); valence band maximum (VBM) is more positive than the oxidation potential of O_2_/H_2_O (1.23 V)].^1^ Despite being stable and often straightforward to synthesize, many oxide photocatalysts have band gaps that are too large for excitation by visible light [e.g., TiO_2_ (3 eV)^2^ and ZnO (3.2 eV^3^)]. On the other hand, although sulfides typically have smaller band gaps, they are often unstable (suffering sulfur self-oxidation) in the catalysis reaction conditions.^4^

One strategy to design new photocatalysts for water splitting under visible light is to consider mixed-anion materials,^5^ and the ability to reduce the band gap by replacing some oxide ions by softer chalcogenide ions (e.g., S^2–^ and Se^2–^) has motivated research into oxychalcogenides for photocatalytic applications. Several p block oxychalcogenides (e.g., Sr_6_Cd_2_Sb_6_O_7_*Q*_10_ (*Q* = S, Se)^6,7^ and LaOInS_2_^8,9^) and d^0^ transition metal oxychalcogenides (Sm_2_Ti_2_S_2_O_5_^10^ and Y_2_Ti_2_O_5_S_2_^11^) have shown promising properties for photocatalytic applications. In an effort to widen the landscape of transition metal oxychalcogenides for photoactive behavior (including photocatalysis and photovoltaicity),^12−14^ we investigated some Fe^2+^ (d^6^) oxychalcogenides to assess their potential for photoactivity, including light-driven water splitting photocatalysis. The quaternary oxychalcogenides CaFeO*Q* adopt layered crystal structures with heteroleptic Fe^2+^ coordination environments and with their polar crystal structures (suggested to enhance electron–hole separation and photocatalytic performance),^15−17^ seemed promising candidates for photoelectrochemical reactions.

**Figure 1:**
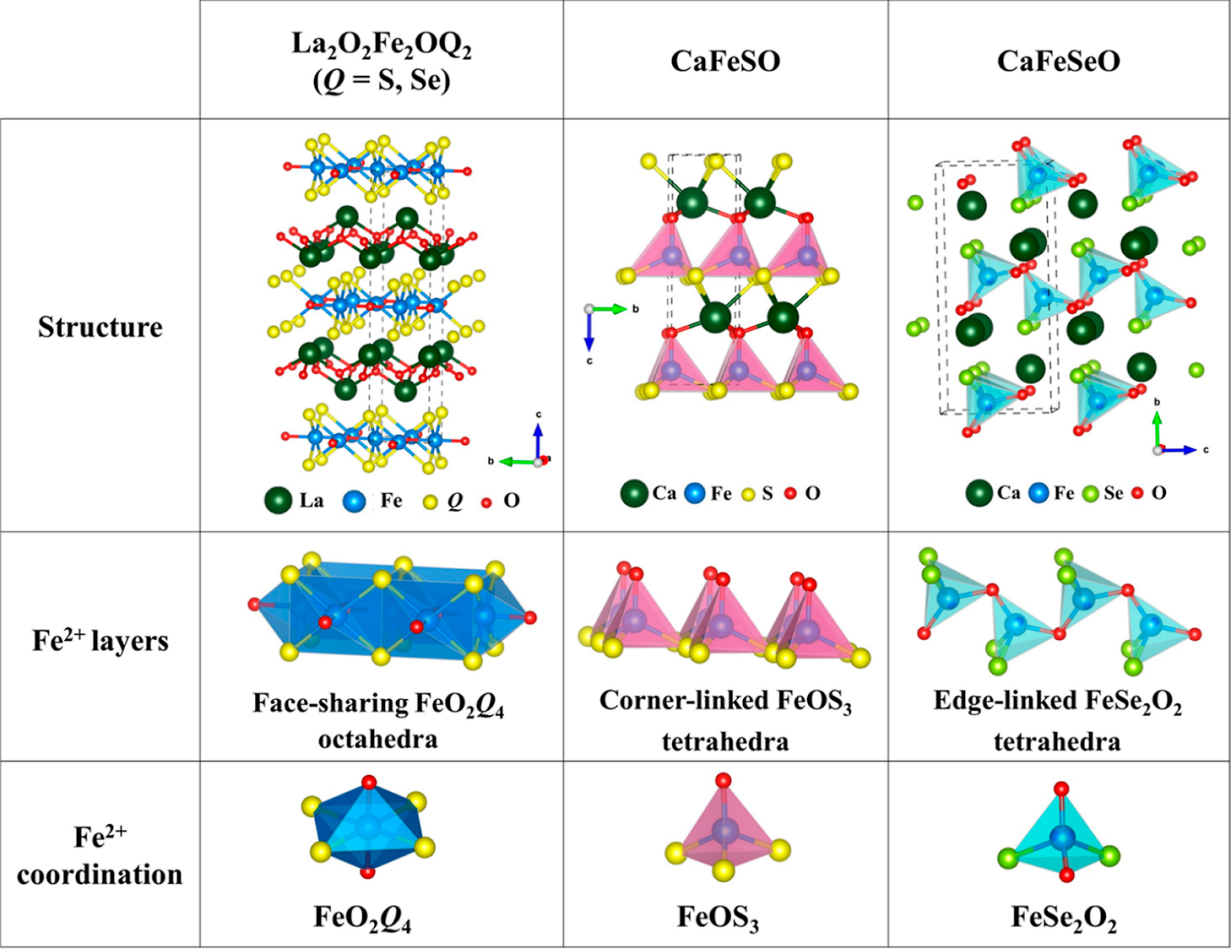
Overview of the crystal structures, Fe^2+^ layers, and coordination environments for La_2_O_2_Fe_2_O*Q*_2_ and CaFeO*Q* (*Q* = S and Se) phases.

CaFeOS crystallizes in a polar, noncentrosymmetric structure of *P*6_3_*mc* symmetry. Its layered structure consists of alternating layers of Ca^2+^ ions and corner-linked FeOS_3_ tetrahedra (Figure 1).^18−20^ These heteroleptic polar units are packed with their dipoles parallel to the hexagonal axis, isostructural with CaZnOS.^21,22^ The photovoltaic activity proposed^23^ for this semiconductor may suggest some promise for photocatalysis. The related oxyselenide CaFeSeO adopts a different structure, composed of puckered layers of corner-linked FeO_2_Se_2_ tetrahedra separated by layers of Ca^2+^ ions (Figure 1).^24,25^ Two polymorphs are known, which differ in the orientation of the polar FeO_2_Se_2_ units: a polar polymorph of *Cmc*2_1_ symmetry with in-plane polarization^25^ and a nonpolar centrosymmetric polymorph of *Pmcn* symmetry.^24^ CaFeOSe is a strongly correlated semiconductor, and the nonpolar polymorph is reported to have an indirect band gap of 1.8 eV.^24^ Our attempts to prepare samples of the nonpolar *Pmcn* polymorph were successful, and so the nonpolar La_2_O_2_Fe_2_O*Q*_2_ phases were used for comparison. They also adopt layered crystal structures but with quite different Fe^2+^ coordination, consisting of face-shared FeO_2_*Q*_4_ octahedra with 180° Fe–O–Fe connectivity.^26^ These Mott-insulating phases have narrow band gaps.^27^ This comparison between CaFeO*Q* and La_2_O_2_Fe_2_O*Q*_2_ phases allows an investigation of the impact of the oxychalcogenide environment around Fe^2+^ cations on the band dispersion and therefore carrier effective masses and mobilities, which are key features for designing photoactive functional materials.

We report here the results of optical and photocurrent measurements on CaFeO*Q* and La_2_O_2_Fe_2_O*Q*_2_ phases and a density functional theory (DFT) calculation of the electronic structure of polar CaFeOSe and its charge carrier effective masses. A photocurrent response was measured for all materials, although the oxysulfide CaFeOS suffers from degradation. The photocurrent response for CaFeSeO indicated fast electron–hole separation, and recombination and transfer rates were calculated for this oxyselenide. Further studies on CaFeO*Q* (*Q* = S or Se) materials to optimize their stability would be interesting for potential photocatalytic materials.

## 2 Methods

La_2_O_2_Fe_2_O*Q*_2_ and CaFeO*Q* (*Q* = S and Se) were prepared by solid–state reactions in evacuated, sealed quartz tubes. Reagents were stored and manipulated in an argon-filled glovebox. For La_2_O_2_Fe_2_O*Q*_2_ (*Q* = S and Se) (0.5 g) analogues, La_2_O_3_, Fe, and S/Se in the molar ratio 2:2.1:2 were used, and the heat treatment consisted of heating to 400 °C (1.5 °C/min) for 12 h and then heating to 600 °C (0.5 °C/min) and then 850 °C for 12 h. For CaFeO*Q* (*Q* = S and Se) (0.5 g) analogues, a mixture of the precursors CaO, Fe, and Se/S in the molar ratio 1:1.05:1 was used. The heat treatment consisted of heating to 750 °C at a rate of 5 °C/min for 60 h before quenching the sample for the oxyselenide and heating to 950 °C (0.5 °C/min) for 24 h for the oxysulfide.

X-ray powder diffraction (XRPD) data were collected on a Bruker D8 A25 diffractometer equipped with a Lynxeye XET linear detector (Cu Kα) in Bragg–Brentano geometry at room temperature with a 1 s counting time and 0.02° step angle. Rietveld refinements using XRPD data were carried out using FullProf software.^28^ The background, sample height, lattice parameters, peak profiles, atomic positions, and atomic displacement parameters were refined. Vesta software^29^ was used to visualize the crystal structure.

The reflectance of the CaFeO*Q* samples was measured from 200 to 900 nm by using a PerkinElmer Lambda 650 spectrophotometer. Diffuse-reflectance UV–visible spectroscopy was used to investigate the magnitude and nature of the band gap of all four phases. After measuring the reflectance vs wavelength, the Kubelka–Munk function^30^ was used to analyze the reflectance data. A Tauc plot [*F*(*R*)*h*υ]^1/*n*^ vs [*h*υ] (where *h*υ is the photon energy and *n* is the type of transition exponent) was used to determine the optical bandgap.^31^

Photocurrent measurements were performed by using an Autolab PGSTAT204 (Metrohm) electrochemical device coupled to a LED module (LED driver kit, Metrohm). The LEDs (450, 470, 505, 530, 590, and 627 nm with low spectral dispersion) were calibrated using a photodiode to determine the density of the luminous flux received by the sample. The photoelectrochemical measurements were performed in a standard three-electrode Magnetic Mount Photoelectrochemical Cell (Redox.me), including Ag/AgCl and Pt wire as reference electrodes and counter electrodes, respectively. The cell allows standardized illumination over 1 cm^2^ by the rear face of the working electrode. The working electrode consisted of the photocatalyst powder dispersed in PVDF (polyvinylidene fluoride) binder (in a 2:1 ratio), which was later deposited on an ITO/glass substrate (Delta Technologies Ltd.) using the drop casting technique.^32^ The electrolyte employed is an aqueous 0.1 M sodium sulfate (Na_2_SO_4_) solution. Mott–Schottky tests were used to determine flat band potentials.^33,34^ Depending on the slope of 1/C^2^ vs applied potential, the flat band potential *E*_fb_ relative to a Ag/AgCl reference electrode or the reversible hydrogen electrode (RHE) can be estimated: *E*_RHE_ = *E*_Ag/AgCl_ + *E*_Ag/AgCl_^0^ + 0.059.pH; where *E*_Ag/AgCl vs SHE_^0^ is the potential of the Ag/AgCl reference electrode with respect to the standard hydrogen electrode (SHE) at 195 mV and the pH of the used electrolyte (5.6 for 0.1 M of Na_2_SO_4_).

The electronic properties of the noncentrosymmetric CaFeOSe oxyselenide were investigated using DFT. Calculations were carried out by employing the projector-augmented-wave (PAW) method^35,36^ encoded in the Vienna ab initio simulation package (VASP)^37^ and the generalized gradient approximation (GGA) of Perdew–Burke–Ernzerhof (PBE)^38^ for the exchange–correlation functionals. To account for the strong electronic correlation associated with the Fe 3d states, the GGA plus on-site repulsion U (GGA + U) method was employed^39^ with *U*_eff_ = 4 eV in an antiferromagnetic configuration.^40^ A plane-wave cutoff energy of 550 eV and a threshold of self-consistent-field energy convergence of 10^–6^ eV were used with k̇-point meshes (13 × 4 × 8) in the irreducible Brillouin zone. It converged with residual Hellman-Feynman forces on the atoms smaller than 0.03 eV Å^–1^ and led to a good match with the experimental structure, i.e., within a reasonable error expected for the GGA method. The relaxed structure was used for calculations of the electronic structure and the charge carrier’s effective masses. The effmass software was used in order to deal with the spin-polarized band structure of the CaFeOSe phase.^41^

## 3 Results

Polycrystalline samples of La_2_O_2_Fe_2_O*Q*_2_ and CaFeO*Q* (*Q* = S and Se) were prepared, and XRPD was used to monitor synthesis reactions. Rietveld analysis (Supporting Information) confirmed the successful preparation of the four phases. Only the noncentrosymmetric, polar polymorph (*Cmc*2_1_ symmetry) of CaFeSeO was prepared; attempts to prepare the nonpolar phase were not successful.

### 3.1 Optical Measurements

**Figure 2:**
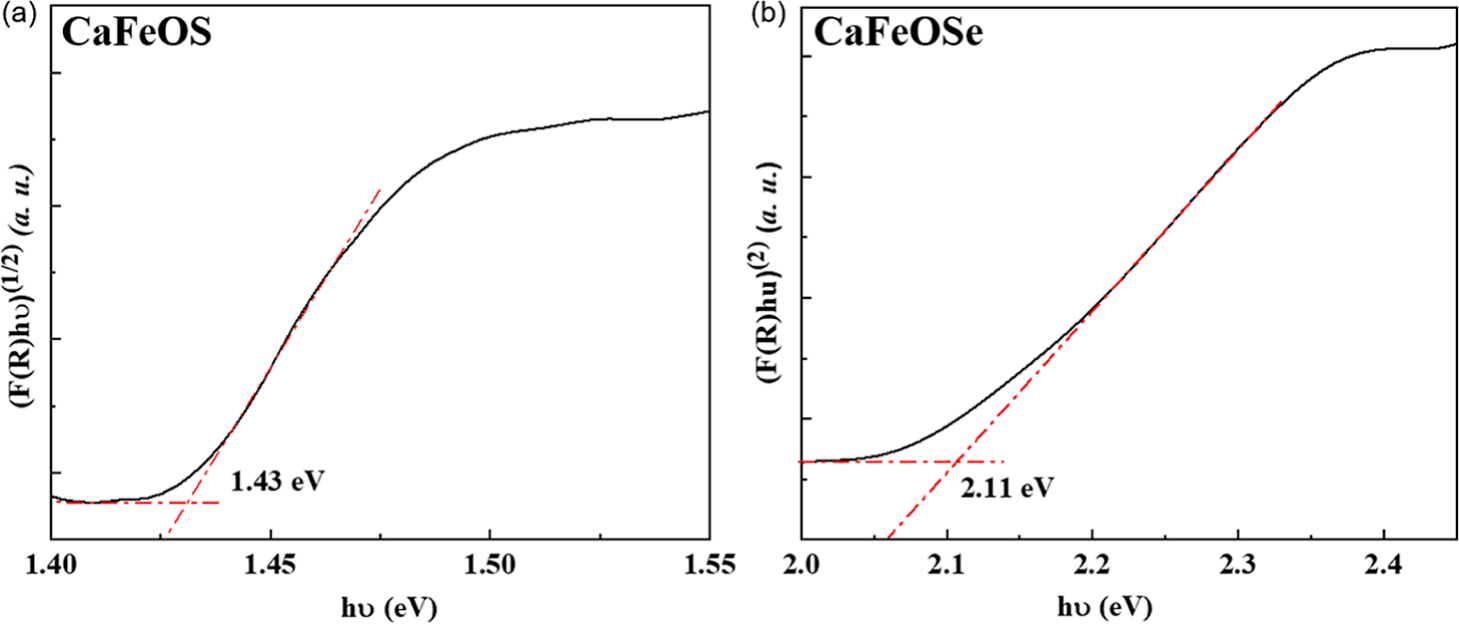
Tauc plots to determine the experimental optical bandgaps for (a) CaFeOS and (b) CaFeOSe.

The band gaps of La_2_O_2_Fe_2_O*Q*_2_ (*Q* = S and Se) are too small to be measured optically, but reported electrical measurements suggest electronic band gaps of 0.19–0.24 eV.^27^ CaFeOS is reported to be an indirect bandgap semiconductor,^23^ while our DFT calculations (see below) indicate that the polar CaFeSeO has a direct gap. Tauc plots^31^ (with *n* = 2 and *n* = 1/2 for CaFeSO and CaFeSeO, respectively) from our diffuse reflectance measurements (after Kubelka–Munk analysis^30^) suggest optical bandgaps of 1.43(1) eV and 2.11(1) eV for CaFeOS and CaFeOSe, respectively (Figure 2). These values are consistent with the literature reports (1.16^23^ and 1.8 eV,^24^ respectively) and are within the energy range of the solar spectrum (1.23–3.1 eV).

In addition to the magnitude of the band gap, the band edge positions of the photoactive materials must also be consistent with the redox reactions of water. The band edge positions were estimated using an empirical method based on Mullikan electronegativities (see Supporting Information), and those for CaFeO*Q* (*Q* = S and Se) were found to be compatible with photocathodic water splitting reactions.

### 3.2 Mott–Schottky Tests

**Figure 3:**
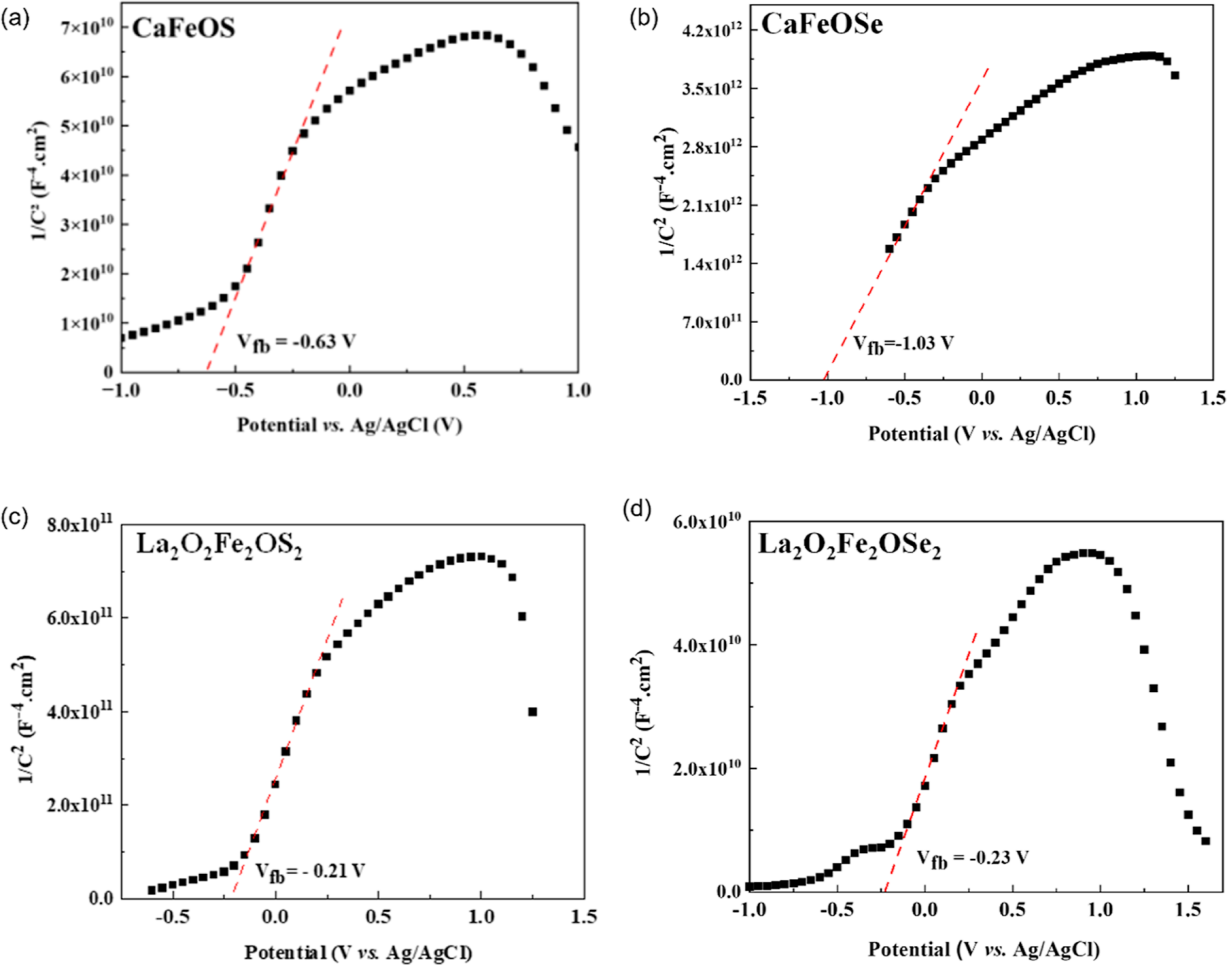
Mott–Schottky plot for (a) CaFeOS, (b) CaFeOSe, (c) La_2_O_2_Fe_2_OS_2_, and (d) La_2_O_2_Fe_2_OSe_2_ deposited on ITO/glass performed at 1 kHz and *V*_bias_ = 0 V.

Mott–Schottky tests were performed for CaFeO*Q* and La_2_O_2_Fe_2_O*Q*_2_ (*Q* = S and Se) at 1 kHz, and zero bias voltage to investigate the conduction type, carrier concentration, flat-band potential *E*_fb_, and plots are shown in Figure 3. The positive slope of  with applied potential confirms the n-type nature of these semiconductors. The *x* axis intercept can be used to determine the flat-band potential with respect to the RHE or a Ag/AgCl reference electrodes (Table 1). These flat-band potentials are close to the CBM^42^ and are consistent with our empirical calculations (Supporting Information) and suggest the potential of the CaFeO*Q* materials for solar water splitting reactions.

**Table 1:** Flat Band Position vs Ag/AgCl and vs RHE

composition	flat band potential (V) vs	
	Ag/AgCl	RHE
CaFeOS	–0.63(1)	–0.105(1)
CaFeOSe	–1.03(1)	–0.505(1)
La_2_O_2_Fe_2_OS_2_	–0.21(1)	0.315(1)
La_2_O_2_Fe_2_OSe_2_	–0.23(1)	0.295(1)

### 3.3 Photocurrent Response

**Figure 4:**
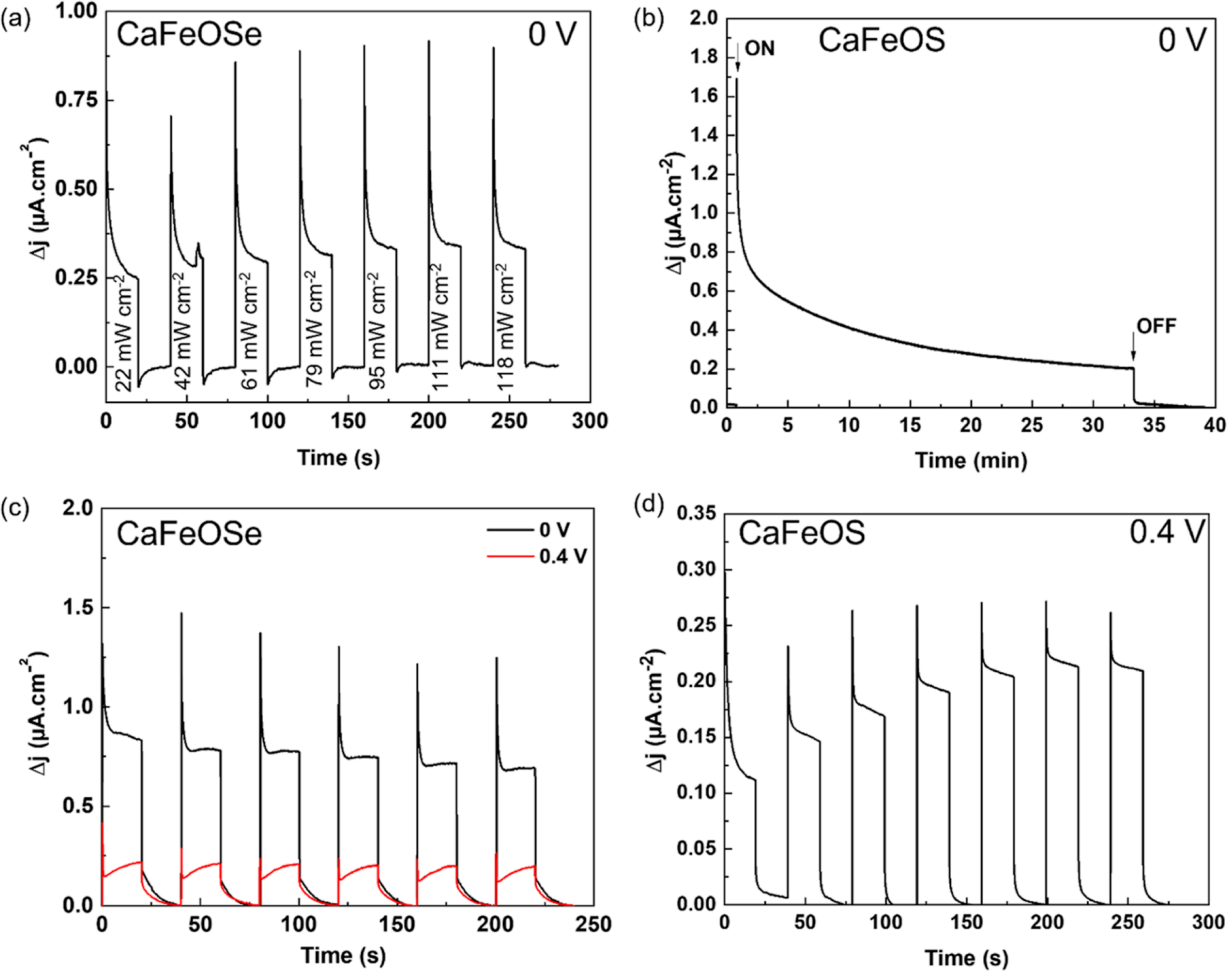
Photocurrent measurements for CaFeSeO: (a) shows transient photocurrent response under several light power densities (450 nm excitation) (*V*_bias_ = 0 V); (b) shows the variation in current density for CaFeOS (*V*_bias_ = 0 V) for >30 min exposure time to solar light excitation; (c) shows transient photocurrent response under solar illumination (100 mW cm^–2^) for *V*_bias_ = 0 and 0.4 V of CaFeOSe; and (d) shows transient photocurrent response under several light power densities (450 nm excitation) for CaFeSeO with *V*_bias_ = 0.4 V.

The greatest photocurrent response (Δ*j* = *j*_illum_ – *j*_dark_, where *j*_illum_ and *j*_dark_ represent the current density under illumination and darkness) was observed for irradiation with 450 nm light for CaFeSeO [with 470 nm light for La_2_O_2_Fe_2_O*Q*_2_ (see Supporting Information)], and so 450 nm light was used for on/off cycles of 20 s to measure the transient photocurrent responses (Figures 4 and 7).

CaFeSeO showed a fairly high photocurrent response (up to 0.9 μA cm^–2^ for a power density of 118 mW cm^–2^) even at *V*_bias_ = 0 V (Figure 4a). The transient photocurrent response shows a spike (charge accumulation at the surface) followed by a decay toward a stable state corresponding to the steady state where the carriers are successfully transferred without undergoing recombination. However, this stable state does not seem to be reached after 20 s of measurement. To verify this, a measurement was carried out over a longer period (Figure 4b), where we observe that this transient state gradually decreases and does not stabilize after 30 min. This evolution could indicate slow kinetics in the establishment of the stationary state, with progressive recombination of electron–hole pairs within the material or a photocorrosion of the electrode (chemical degradation at the interface of the film or progressive dissolution in the electrolyte). The first hypothesis, of slow kinetics, seems more likely because the intensity of the photocurrent remains relatively stable after several ON/OFF cycles under solar irradiation (Figure 4c). Additionally, trap states in the photoconductor can play an important role in extending the lifetime of photogenerated carriers. Thus, the long decay may be due to intrinsic defects (such as impurities, vacancies, or interstitial ions), which induce energy levels in the band gap.^43,44^ The recombination phenomena are quite rapid, but if the semiconductor contains traps, the establishment of the steady state can be slower with the presence of shallow traps (close to band edges) or even slower with the presence of deep traps (close to the middle of the band gap).^45^ For an applied bias potential of 0.4 V, the behavior evolves with a lower peak height (Figure 4d), indicating a decrease in the recombination rate.

**Figure 5:**
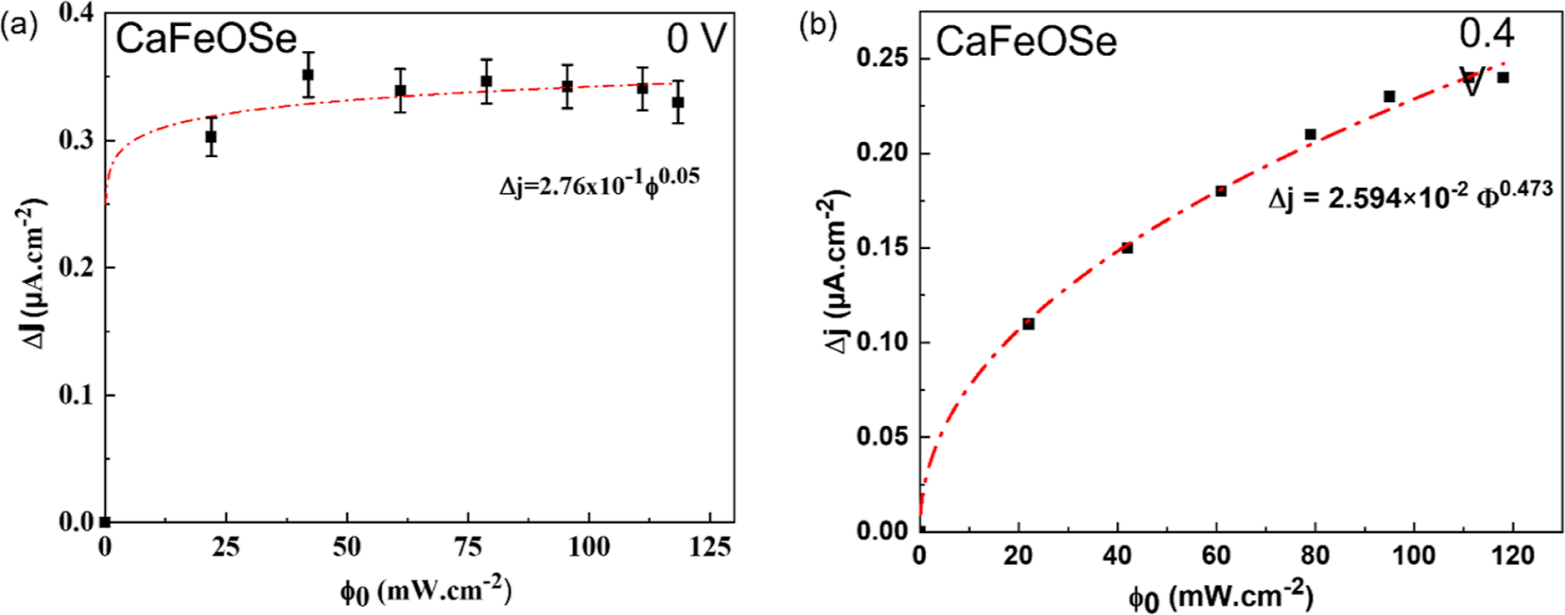
Evolution of the photocurrent density for CaFeOSe with the power density of light under a 450 nm excitation for (a) *V*_bias_ = 0 V and (b) *V*_bias_ = 0.4 V.

In addition, the photocurrent response of CaFeSeO was found to increase as a function of the power density of light (Figure 5). This behavior could be fitted by a classical power law.^46^ For *V*_bias_ = 0 V and Δ*j* = 2.76 × 10^–1^(2)Φ^0.05(2)^, the low exponent from this fitting indicates that a saturation regime is quickly reached after 20 s of measurement. Thus, increased illumination power cannot effectively increase the photogain.^47^ (The slow recombination kinetics described above are not taken into account in this case because the steady state is not reached.) For *V*_bias_ = 0.4 V, the power law follows a more usual evolution with Δ*j* = 2.59 × 10^–2^(2)Φ^0.47(2)^, indicating faster detrapping with the application of a bias voltage.

**Figure 6:**
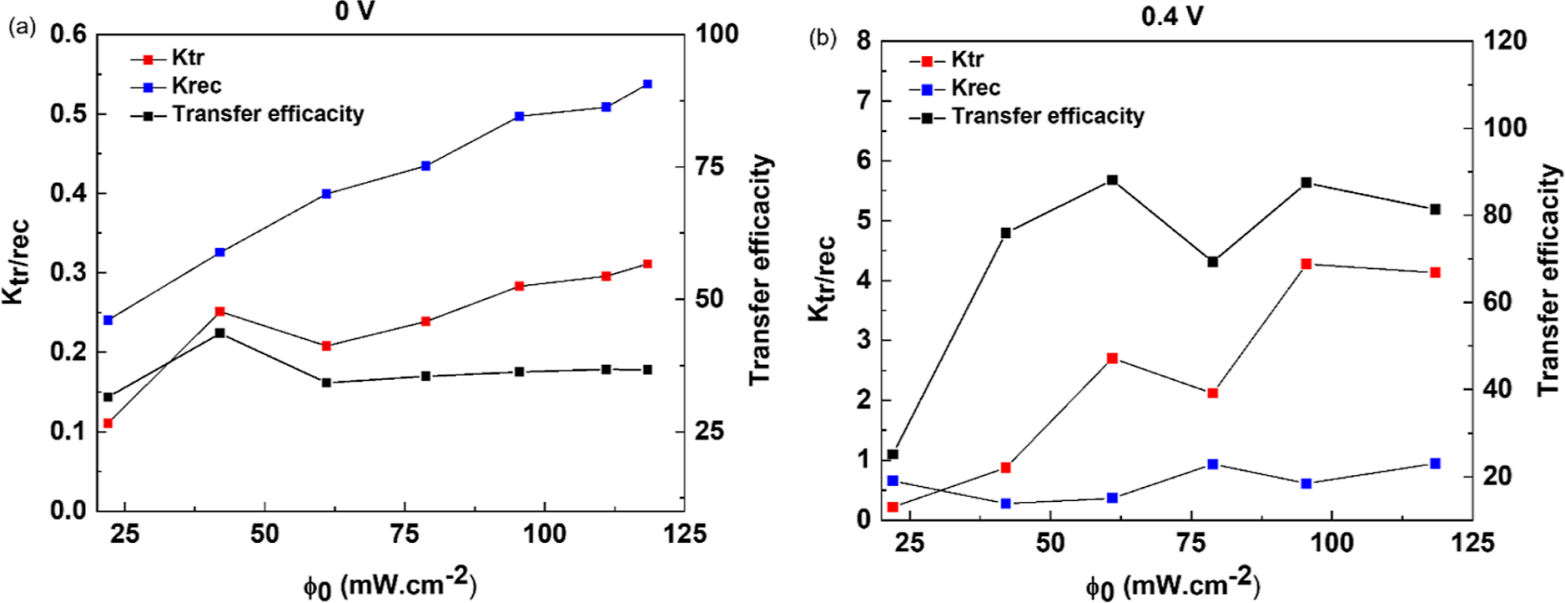
Evolution of the recombination and transfer rate constants *k*_tr_ and *k*_rec_ with intensity of light alongside the transfer efficacity η_k_ by intensity light of CaFeOSe at (a) 0 and (b) 0.4 V.

The characteristic shape of the photocurrent response observed for CaFeSeO (Figure 4a) indicates the fast separation of charge carriers (the peak results from the surface being loaded with charge carriers), followed by the system reaching an equilibrium between charge recombination and charge transfer (the decay from the spike to the plateau at steady state).^48^ The exponential decrease in the photocurrent from the peak to the plateau can be fitted using a model proposed by Parkinson et al.^49^ to give values for the transfer and recombination rate constants (Figure 6). For *V*_bias_ = 0 V, *k*_rec_ increases monotonically (*k*_rec_ = 0.24 to 0.55 min^–1^ for 21 to 118 mW cm^–2^) with an increase in ϕ_0_ indicating that the recombination of electrons and holes is favored under high light power density (as described for WO_3_ photoanodes).^50^ The transfer rate remains lower than the recombination rate, leading to a transfer efficiency of 40%. In contrast, for *V*_bias_ = 0.4 V, the transfer rate is greater than the recombination rate (the recombination rate remains stable at around *k*_rec_ = 0.5 min^–1^) giving an improvement in transfer efficiency of up to 80% (Figure 6b). These rate constants for *V*_bias_ = 0.4 V calculated for CaFeSeO are noticeably higher than those measured recently for Sr_6_Cd_2_Sb_6_S_10_O_7_ (*k*_tr_ = 0.25 min^–1^ and *k*_rec_ = 0.08 min^–1^).^6^ The application of potential therefore promotes the transfer of charge at the interface.

The transient photocurrent response of CaFeSeO under solar illumination (simulated using a 150 W xenon lamp with an AM 1.5G filter, 100 mW cm^–2^) was also measured for *V*_bias_ = 0 and 0.4 V for on/off cycles of 20 s (Figure 4c). Apart from the good reproducibility of the measurements over the different cycles, two behaviors are observed. First, for *V*_bias_ = 0 V with high photocurrents (1.45 μA cm^–2^), the steady state is not reached within the measurement time. Second, for *V*_bias_ = 0.4 V, a more stable but lower photocurrent (0.35 μA cm^–2^) is measured.

For CaFeSO, no photocurrent response was detected for *V*_bias_ = 0.0 V. It is necessary to apply a voltage of 0.6 V in order to observe less stable and much weaker photocurrents [∼40 nA cm^–2^ for a power density of 111 mW cm^–2^ (450 nm), see Supporting Information] compared with the oxyselenide analogue, demonstrating poor performance of this material. Furthermore, for higher potentials, the response is erratic until it completely disappears, indicating strong photocorrosion of the film. This could result from some degradation of the CaFeSO film, particularly under the higher bias voltage of *V*_bias_ = 0.6 V. Linear sweep measurements (see Supporting Information) give evidence of an oxidation reaction for the sample-electrolyte system, likely indicating that some oxidation of Fe^2+^ in the sample occurs.

**Figure 7:**
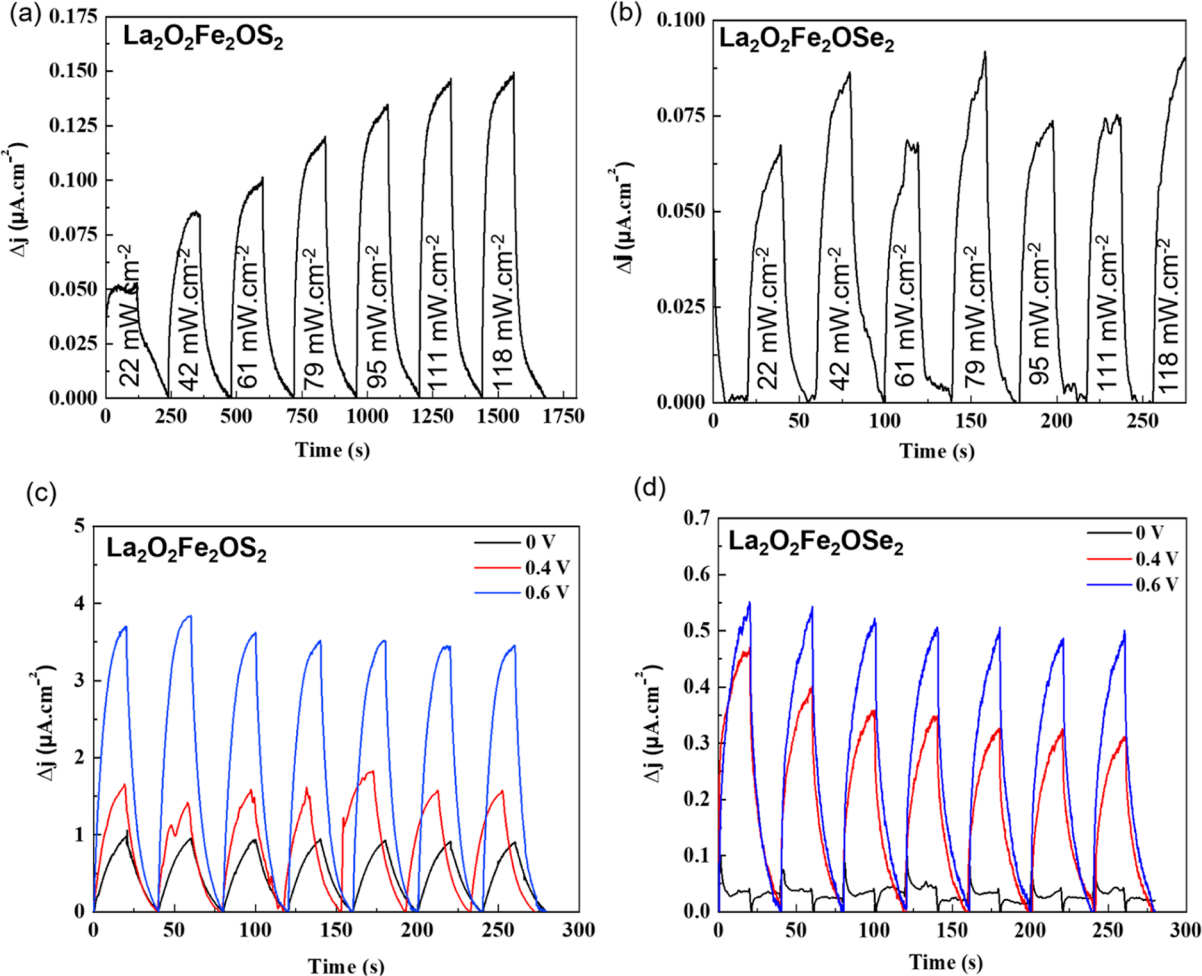
Transient photocurrent response under a 450 nm excitation of (a) La_2_O_2_Fe_2_OS_2_ and (b) La_2_O_2_Fe_2_OSe_2_ and under solar illumination (100 mW cm^–2^) for *V*_bias_ = 0, 0.4, and 0.6 V of (c) La_2_O_2_Fe_2_OS_2_ and (d) La_2_O_2_Fe_2_OSe_2_.

Similar photocurrent response measurements for La_2_O_2_Fe_2_O*Q*_2_ showed a stable photocurrent (up to 0.15 μA cm^–2^) for *Q* = S (Figure 7a), and the study of the transient photocurrent response over a longer time (see Supporting Information) shows good stability with only a very slight decrease over >30 min. An unstable and lower (up to 0.08 μA cm^–2^) photocurrent was measured for *Q* = Se (Figure 7b). The transient current has slower kinetics for the oxyselenide phase, as demonstrated by the faster exponential growth for the oxysulfide (Figure 7c,d). The transient photocurrent responses were also measured for La_2_O_2_Fe_2_O*Q*_2_ under solar illumination (simulated using a 150 W xenon lamp with an AM 1.5G filter and 100 mW cm^–2^) for *V*_bias_ = 0, 0.4, and 0.6 V (Figure 7c,d). As expected, the measured photocurrent increased with increasing *V*_bias_, although a slight decrease in photocurrent with time was observed under the applied voltage, possibly indicating some photocorrosion (chemical degradation or dissolution of the electrode in the electrolyte), which seems to be more significant for La_2_O_2_Fe_2_OSe_2_. The evolution of the photocurrent response with power density was also measured for La_2_O_2_Fe_2_OS_2_ (see Supporting Information) and showed behavior consistent with a much high exponent (0.60(2)) than that determined for CaFeOSe (Figure 4d), indicating fewer traps for La_2_O_2_Fe_2_OS_2_ than for the other oxychalcogenides discussed here. This evolution of the photocurrent according to the luminous flux indicates the potential of La_2_O_2_Fe_2_OS_2_ for photodetector applications.

### 3.4 Electronic Structure

**Figure 8:**
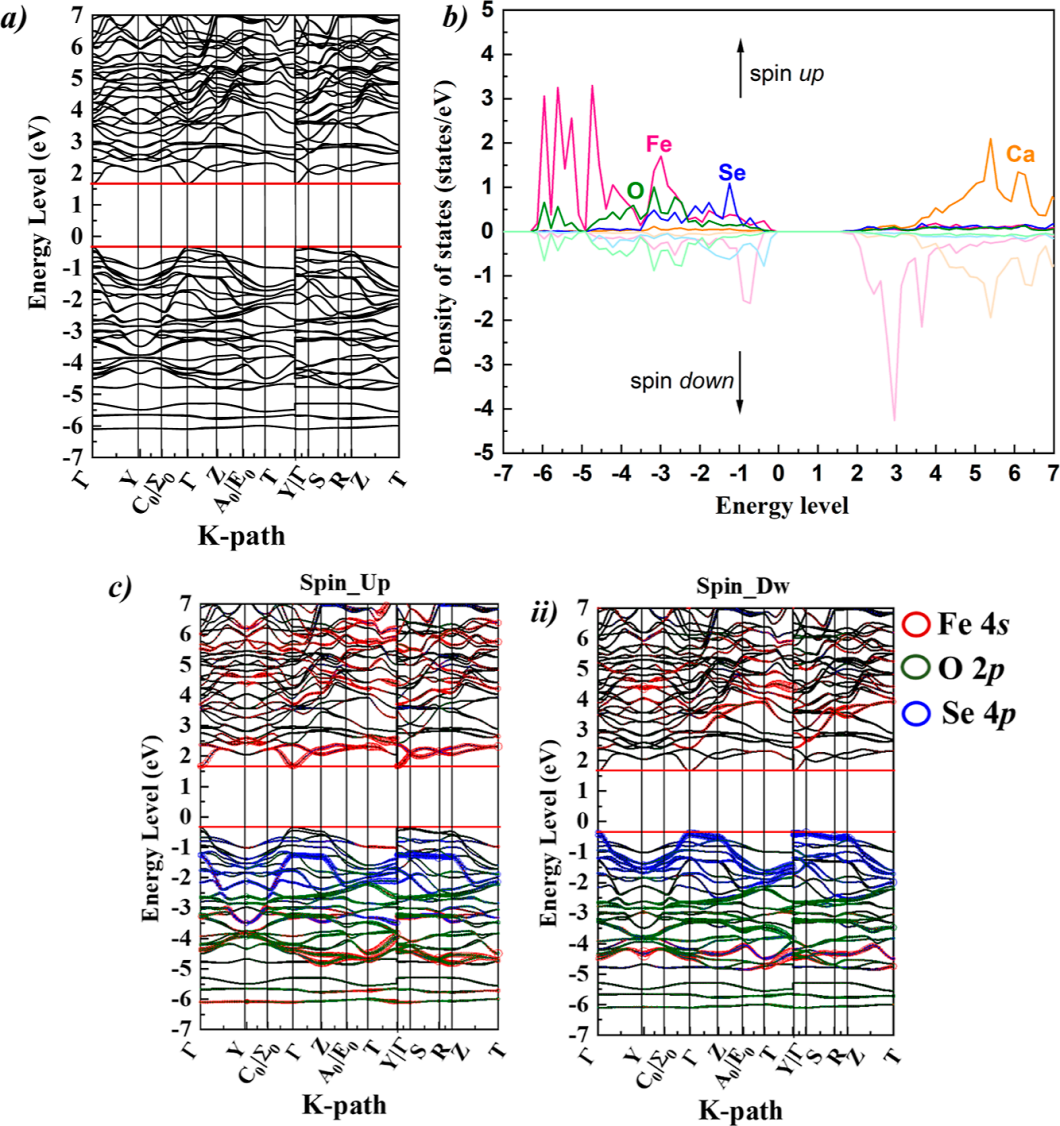
DFT calculations of the noncentrosymmetric (*Cmc*2_1_) polymorph of CaFeOSe with (a) electronic band structure, (b) PDOS, and (c) fat bands showing the Fe 3d states in (i) spin up and (ii) spin down.

The band structure and projected density of states (PDOS) were calculated for the polar polymorph of CaFeOSe studied here (Figure 8), for comparison with the electronic structures reported for CaFeOS and for La_2_O_2_Fe_2_OQ_2_.^27^ Our calculations suggest a direct band gap of 2.08 eV for the polar polymorph of CaFeOSe (consistent with our optical measurements, Figure 2), in contrast to the indirect nature reported for the nonpolar polymorph.^24^ The Fe 3d states dominate the bottom of the conduction band and also hybridize with the O 2p and Se 3p states to form the top of the valence band (Figure 8b). This is comparable with the PDOS reported for the nonpolar polymorph of CaFeOSe,^24^ and qualitatively similar to that reported for CaFeOS.^23^ Analysis of the fat band of the Fe orbitals and of their PDOS (spin up and down) in Figure 8b,c indicates a high spin state.

The band dispersions can provide insight into the carrier mobilities. Different dispersions at the CBM and VBM suggest different mobilities of the electrons and holes. The lowest electron effective mass *m*_e_* = 0.342(3) m_0_ was found for the electrons in the conduction band for the Γ → S direction (i.e., within the layers), while the hole effective mass was heavier (*m*_h_* = 3.616(3) m_0_) along this direction. This indicates a high intralayer mobility for the electrons (*m*_e_* < 0.5 m_0_) and lower mobility for the holes. This is consistent with computational work suggesting that having s orbital character at the CBM (the Fe 4s contribution to the spin-up channel, Figure 8c) can give low effective masses.^51^

## 4 Discussion

The iron oxychalcogenides investigated here share common features, including their layered crystal structures (Figure 1) and the mixed-anion coordination environments of Fe^2+^ cations (FeO_2_*Q*_4_ for La_2_O_2_Fe_2_O*Q*_2_, FeOS_3_ for CaFeOS, and FeSe_2_O_2_ for CaFeOSe). These features allow us to explore structure–property relationships in the context of photocatalysis for this family of materials.

**Figure 9:**
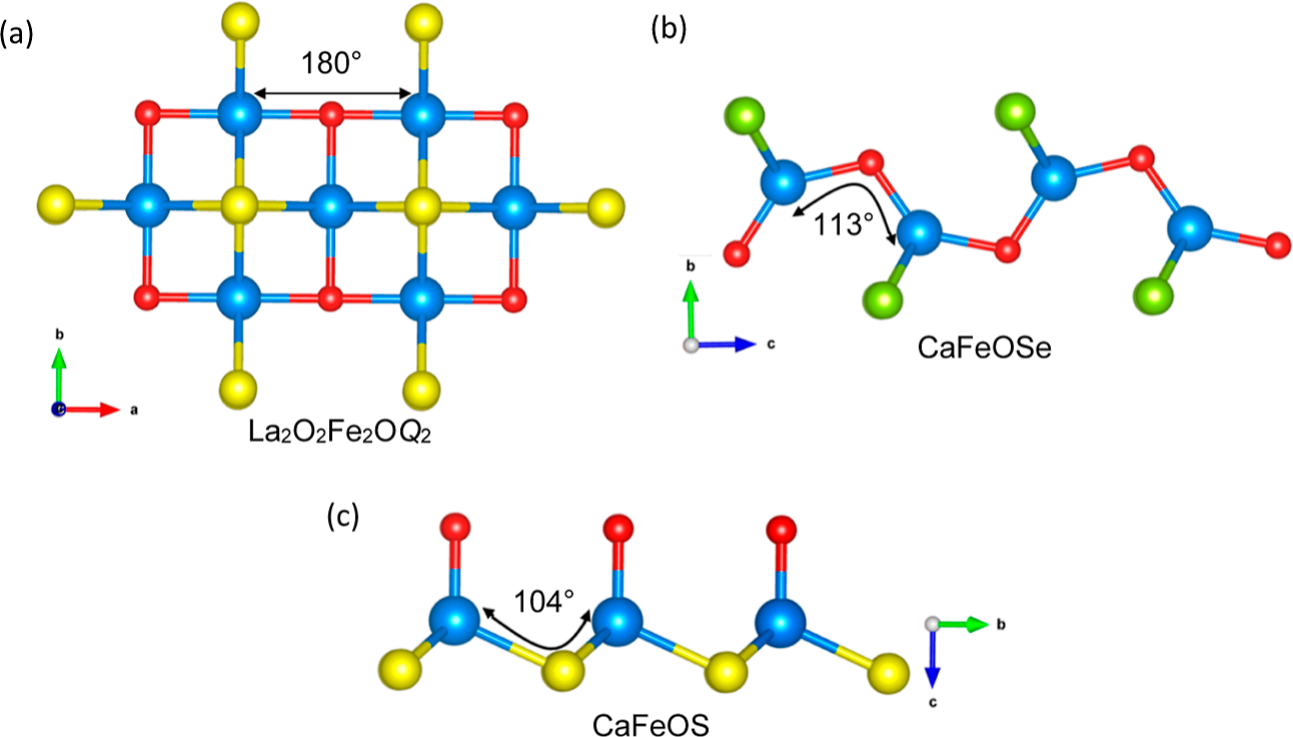
Representation of the M-O/*Q*-M bond angles in (a) La_2_O_2_Fe_2_O*Q*_2_ (*Q* = S and Se), (b) CaFeOSe, and (c) CaFeOS.

Both CaFeSeO and CaFeSO have band gaps well-matched to the solar spectrum (1.43(1) and 2.11(1) eV for *Q* = S and Se, respectively, Figure 2). This contrasts with the Fe^2+^ oxide CaFeO_2_ (composed of puckered FeO_4_ square planar units) with a much larger band gap of ∼2.7 eV.^52−54^ DFT studies on CaFeO*Q* (*Q* = S and Se) suggest that the VBM and CBM are predominantly composed of Fe 3d states but with significant hybridization with *Q* np states,^23,24^ presumably contributing to the wider bands and the reduced band gap in these oxychalcogenides. The reduction in bandgap for CaFeOS compared with CaFeOSe is likely due to the increased ratio of chalcogenide to oxide in the pseudotetrahedral Fe coordination environment (FeS_3_O units in the oxysulfide compared with FeSe_2_O_2_ units in the oxyselenide)^55^ and the effect of chemical pressure with the smaller sulfide anion.^56^ The much smaller bandgaps for La_2_O_2_Fe_2_O*Q*_2_ reflect the different Fe environments and connectivity in these Mott insulators: the 180° Fe–O–Fe bond angles (Figure 9) give better orbital overlap and more dispersed bands, contributing to the small band gaps in these materials.^27^ This contrasts with CaFeO*Q* phases with 113° Fe–O –Fe and 104° Fe–S–Fe bond angles connecting Fe-centered tetrahedra for *Q* = Se and S, respectively, giving flatter bands and wider band gaps (Figure 8^23^).

Both La_2_O_2_Fe_2_OQ_2_ and CaFeO*Q* (*Q* = S and Se) phases generated reproducible photocurrents under solar irradiation and over the whole visible spectrum range. The spike observed in the transient photocurrent response for CaFeSeO indicates fast carrier generation (e^–^–h^+^ separation), then the establishment of a steady state with a balance between transfer and recombination phenomena, notably at the surface of the sample (Figure 4). It has been shown that an internal field due to a polar crystal structure (e.g., in ferroelectrics) minimizes charge carrier recombination and instead favors transfer at the interfaces.^57^ Both CaFeSeO and CaFeSO samples studied in this work adopt polar crystal structures (of *Cmc*2_1_ and *P*6_3_*mc* symmetries, respectively)^18,25^ and are composed of polar units (FeO_2_Se_2_ and FeOS_3_ pseudotetrahedra), in contrast to the centrosymmetric, nonpolar structures of La_2_O_2_Fe_2_O*Q*_2_ (*I*4/*mmm* symmetry) with slower kinetics. It is not clear whether a dipole across the photoactive cation or a polar axis in the crystal structure would have the greater effect of enhancing e^–^–h^+^ separation. Comparison with LaGaS_2_O (with a nonpolar structure of *Pbcm* symmetry but composed of polar GaO_2_S_2_ units),^58^ which shows a qualitatively similar photocurrent response with fast e^–^–h^+^ separation,^59^ suggests that the local polarity of the photoactive units might be more significant than the overall polarity of the crystal structure. Similar studies on the nonpolar polymorph of CaFeSeO^24^ would be interesting to confirm this. It is interesting that a higher photocurrent was observed for CaFeOSe with *V*_bias_ = 0.0 V (1.45 μA cm^–2^) compared with *V*_bias_ = 0.4 V (0.35 μA cm^–2^). This could be explained by some film degradation in the applied voltage. Further investigations are needed to understand this behavior.

The very different photochemical behavior of CaFeOSe and CaFeOS (Figure 4 and Supporting Information) results from the oxidative degradation of CaFeOS (at *V*_bias_ = 0.6 V). This illustrates that the stability of the photoactive oxychalcogenide is an important challenge to overcome in developing this family of materials. Lower oxidation states and coordination numbers can often be stabilized in oxychalcogenides compared with typical oxides,^60^ but this can leave the transition metal susceptible to oxidation, depending on the conditions. The greater stability of CaFeOSe here might be due to the greater concentration of electronegative oxide ions in the FeO_2_Se_2_ units stabilizing the Fe^2+^ cation compared with the FeOS_3_ units in CaFeOS. It has been reported that holes in d bands of transition metal dichalcogenides might react quite differently to holes in p bands of p block chalcogenides,^61^ suggesting that further research might be needed to understand the different stabilities of p block vs transition metal oxychalcogenides in conditions for photoelectrochemical reactions. Related to this, the surface morphology of these samples could also play a key role in their performance and stability. Surface states (associated with dangling bonds at surfaces exposed to the electrolyte) can be detrimental to performance, acting as charge recombination centers^62^ or conversely under appropriate irradiation, they can act as electron donors, giving a photocurrent response.^63^ Studies on the surface morphology and modification (as carried out with ferrites)^64,65^ would be useful to optimize the stability and performance of CaFeOSe.

## 5 Conclusions

The structural and physical properties of four iron-based oxychalcogenides, La_2_O_2_Fe_2_O*Q*_2_ and CaFeO*Q* (*Q* = S and Se), were investigated by exploring their photoelectrochemical and electronic characteristics to determine their potential as photocatalysts. The optical band gaps of CaFeO*Q* (*E*_g_ = 1.43(1) and 2.11(1) eV for *Q* = S and Se, respectively) and conduction band edge positions were found to be suitable for half reactions in visible light as photocathodes. The band gaps of Mott-insulating La_2_O_2_Fe_2_O*Q*_2_ (*Q* = S and Se) were too small for photocatalytic activity. The transient photocurrent response of CaFeOSe shows spikes (Figure 4), indicating very efficient electron–hole separation and migration, consistent with effective masses calculated by DFT. The O/*Q* ratio in the Fe^2+^ coordination environment in CaFeOSe to give O-linked FeO_2_Se_2_ tetrahedra seems to reflect a balance between stabilizing the Fe^2+^ cation (in contrast to CaFeOS, which was oxidized by the electrolyte) and reducing the band gap to match the visible spectrum. Further work to investigate the role of mixed-anion environments in tuning the band gap, stability, and polarity of coordination environments and the balance between these for optimal performance would give important insights for designing new photoactive materials, including photocatalysts with activity under solar irradiation.

## Supporting Information Available

Rietveld refinement details of La_2_O_2_Fe_2_O*Q*_2_ and CaFeO*Q* (*Q* = S and Se) using room temperature XRPD data; band edges positions; and electrochemical additional measurements (PDF)
