# Supplementary material for: Iron Oxychalcogenides and Their Photocurrent Responses
Source: Inorg Chem. 2024 Feb 2;63(7):3292–302. doi: 10.1021/acs.inorgchem.3c03672 (PMC10880050; doi:10.1021/acs.inorgchem.3c03672)
Supplement: Supplementary file 1 — ic3c03672_si_001.pdf [file ic3c03672_si_001.pdf]

Supporting information:
Iron oxychalcogenides and their photocurrent responses

Sandy Al Bacha[a,b,c], Sébastien Saitzek[d], Houria Kabbour[a]*, Emma E. McCabe[c]*

[a] Univ. Lille, CNRS, Centrale Lille, ENSCL, Univ. Artois, UMR 8181 – UCCS – Unité de Catalyse et Chimie du Solide, F-59000 Lille, France
[b] University of Kent, School of Physical Sciences, Canterbury, Kent CT2 7NH, U.K
[c] Durham University, Departement of Physics, Durham DH1 3LE, U.K.
[d] Univ. Artois, CNRS, Centrale Lille, Univ. Lille, UMR 8181, Unité de Catalyse et Chimie du Solide (UCCS), F-62300 Lens, France.

Corresponding authors: houria.kabbour@univ-lille.fr ; emma.mccabe@durham.ac.uk

Supporting information:
1) Synthesis and preliminary characterisation of CaFeO*Q* and $La_2O_2Fe_2OQ_2$ samples

a)

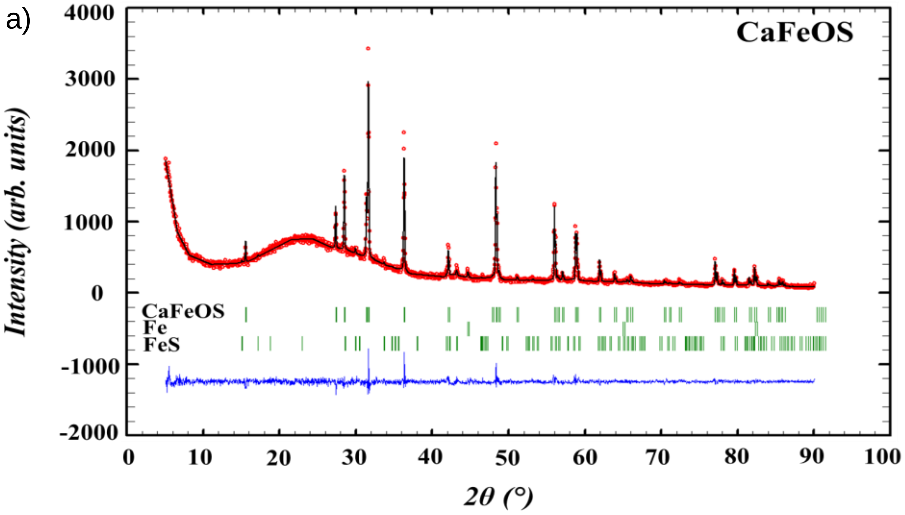

b)

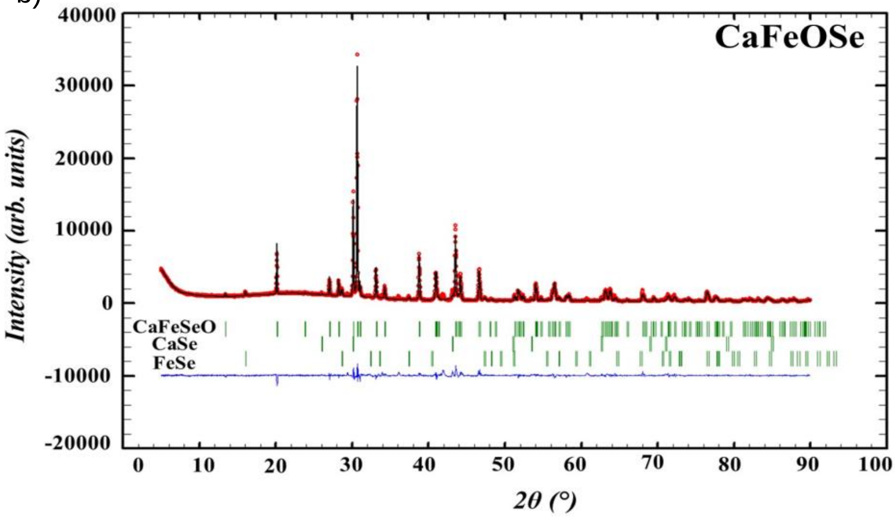

c)

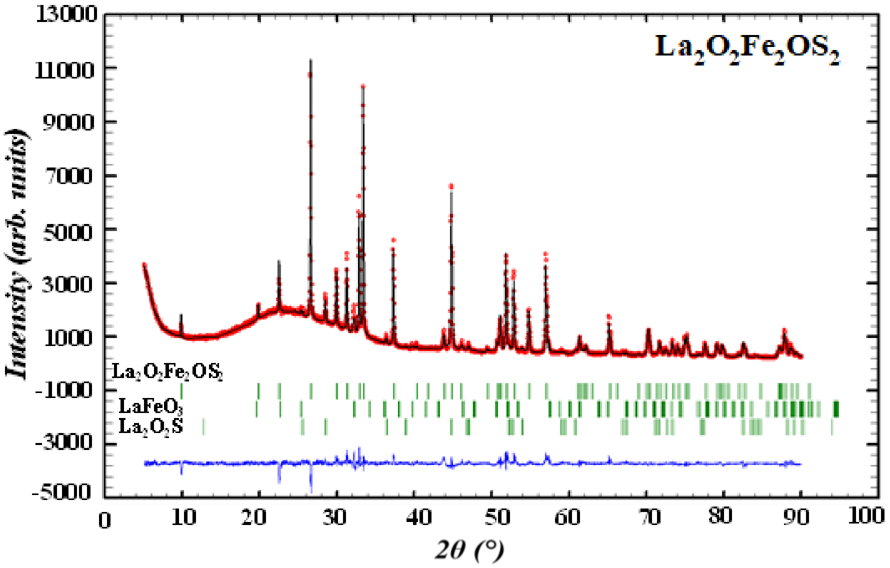

d)

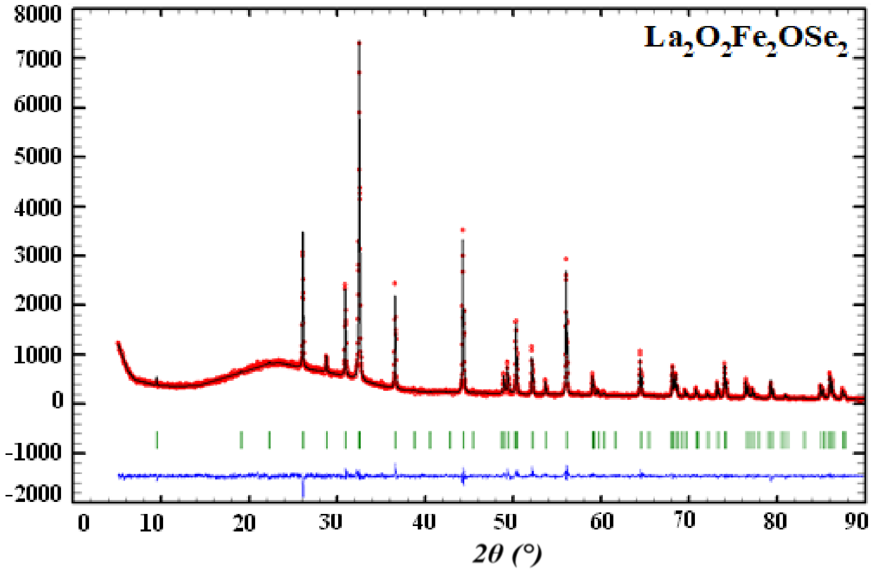

Figure SI1 Powder XRD Rietveld refinement profiles: the experimental (red) and the calculated (black) patterns are superimposed; the difference curve and Bragg positions are shown in blue and green, respectively of (a) CaFeOS (95.59%), Fe (0.71%), FeS (3.70%) and (b) CaFeOSe (93.30%), CaSe (2.06%), FeSe (4.65%); (c) $La_2O_2Fe_2OS_2$ (88.49%), $LaFeO_3$ (7.30%), La_2O_2S (4.20%) and (d) $La_2O_2Fe_2OSe_2$.

Table SI1 Refinement details from Rietveld refinement using room temperature XRPD data.

		CaFeOS	CaFeOSe	$La_2O_2Fe_2OS_2$	$La_2O_2Fe_2OSe_2$
**Space group**		$P6_3mc$	$Cmc2_1$	*I4/mmm*	*I4/mmm*
**Unit cell parameters**	***a*** **(Å)**	3.7613(1)	3.8925(1)	4.0438(1)	4.0862(1)
	b **(Å)**	3.7613(1)	13.1878(2)	4.0438(1)	4.0862(1)
	c **(Å)**	11.3893(2)	5.9260(1)	17.8995(3)	18.6028(2)
	V **($Å^3$)**	139.54(1)	304.20(1)	292.70(1)	310.61(1)
**Fitting statistics**	χ^2	1.49	9.02	3.86	1.80
	R_{exp} **(%)**	5.91	3.39	3.32	5.43
	R_{wp} **(%)**	7.20	10.2	6.53	7.28
	R_p **(%)**	5.47	7.16	4.70	5.43

2) Estimated band edge positions for $La_2O_2Fe_2OQ_2$ and CaFe*Q*O

The band edge positions for $La_2O_2Fe_2OQ_2$ and CaFe*Q*O (Q = S, Se) were estimated using an empirical method[1-3] based on Mullikan electronegativities using equation 1:

$$E_{VB,CB} = E_0 + \prod_{M=1}^{n} \chi_M^{j\ 1/n} \pm E_{gap}/2 \qquad (1)$$

where $E_{VB,CB}$ corresponds to the positions of the valence and conduction band edges, E_0 to the difference bwrween NHE and the vacuum ($E_0 = -0.45$ eV), χ_M is the atom M's electronegativity on the Mulliken scale with n the number of atoms and j the stoichiometric ratio. The results are summarized in Table SI2.

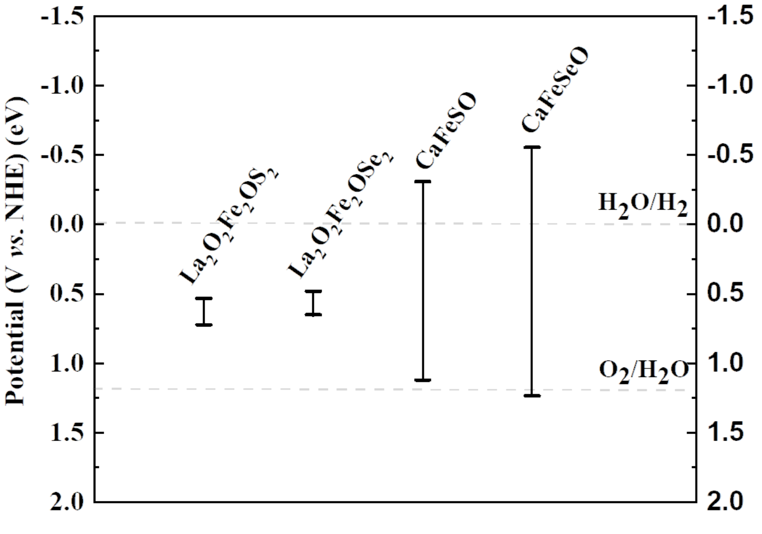

Figure SI2 Calculated band edges positions for CaFeOS and CaFeOSe. The levels of H_2 and O_2 evolution are indicated by dashed lines.

Table SI2 Optical bandgap and the relative band edges position.

Composition	Optical bandgap (eV)	Band edges position	
		CB	VB
**CaFeOS**	1.43(1)	-0.31(2)	1.12(2)
**CaFeOSe**	2.11(1)	-0.71(2)	1.4(1)
**$La_2O_2Fe_2OS_2$** [4]	0.24	0.53(2)	0.72(2)
**$La_2O_2Fe_2OSe_2$** [4]	0.19	0.48(2)	0.65(2)

Both the magnitude of the optical band gaps and the band edge positions show the potential of CaFeO*Q* (*Q* = S, Se) for photocathodic solar water splitting reactions whilst $La_2O_2Fe_2OQ_2$ (*Q* = S, Se) are unlikely to be photocatalytically active for water splitting reactions.

3) Photocurrent measurements of CaFe*Q*O samples

CaFeSeO absorbs in the visible spectrum (400 – 700 nm), consistent with the optical bandgap measured by diffuse reflectiance (Figure 2). The photocurrent response was measured for a range a wavelengths (for $V_{bias} = 0, 0.4$ V, Figure SI3) and the highest response was observed for 450 nm irradiation.

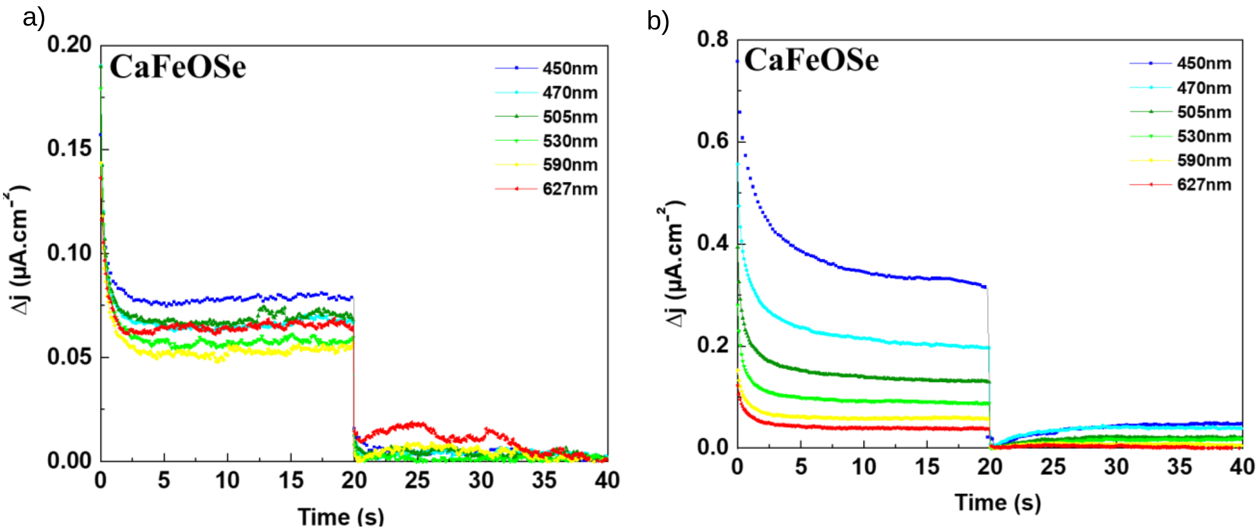

Figure SI3 Transient photocurrent response *vs.* wavelengths (constant light intensity ϕ_0=42 mW cm^{-2}) for CaFeSeO with (a) $V_{bias} = 0.4$ V and (b) $V_{bias} = 0.0$ V.

4) Oxidation of CaFeSO

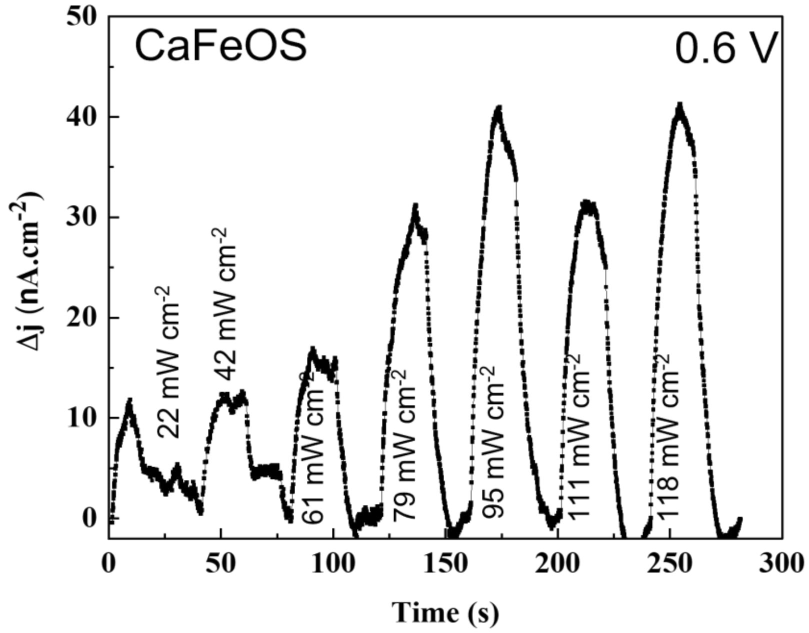

Figure SI4 Transient photocurrent under several light power densities (450 nm) of CaFeSO at V_{bias}= 0.6 V

A linear sweep measurement was carried out on CaFeSO (in 0.1 M Na_2SO_4 electrolyte) (Figure SI5). An oxidation reaction is observed for V ≥ 0.7 V, consistent with the redox potential of Fe^{2+}/Fe^{3+} and the oxidation reaction $Fe^{2+} \rightleftharpoons Fe^{3+} + e^-$. This oxidative degradation of the CaFeSO material likely explains the poor photocurrent response measured.

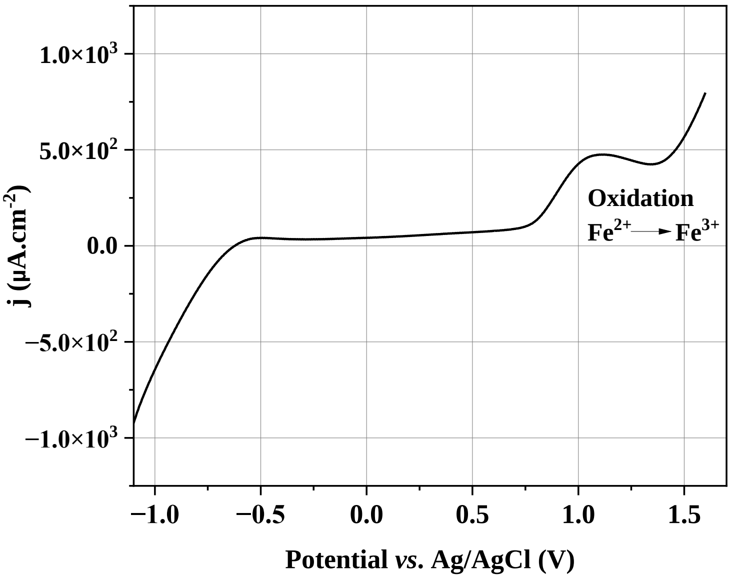

Figure SI5 Linear sweep plot for CaFeOS.

5) Photocurrent measurements of $La_2O_2Fe_2OQ_2$ samples

$La_2O_2Fe_2OQ_2$ have much smaller band gaps with absorption spanning the IR range (700 nm – 1 mm) and so can also absorb higher energy light from the visible range. Maximum photocurrents were measured at 470 nm for both these phases (Figure SI6).

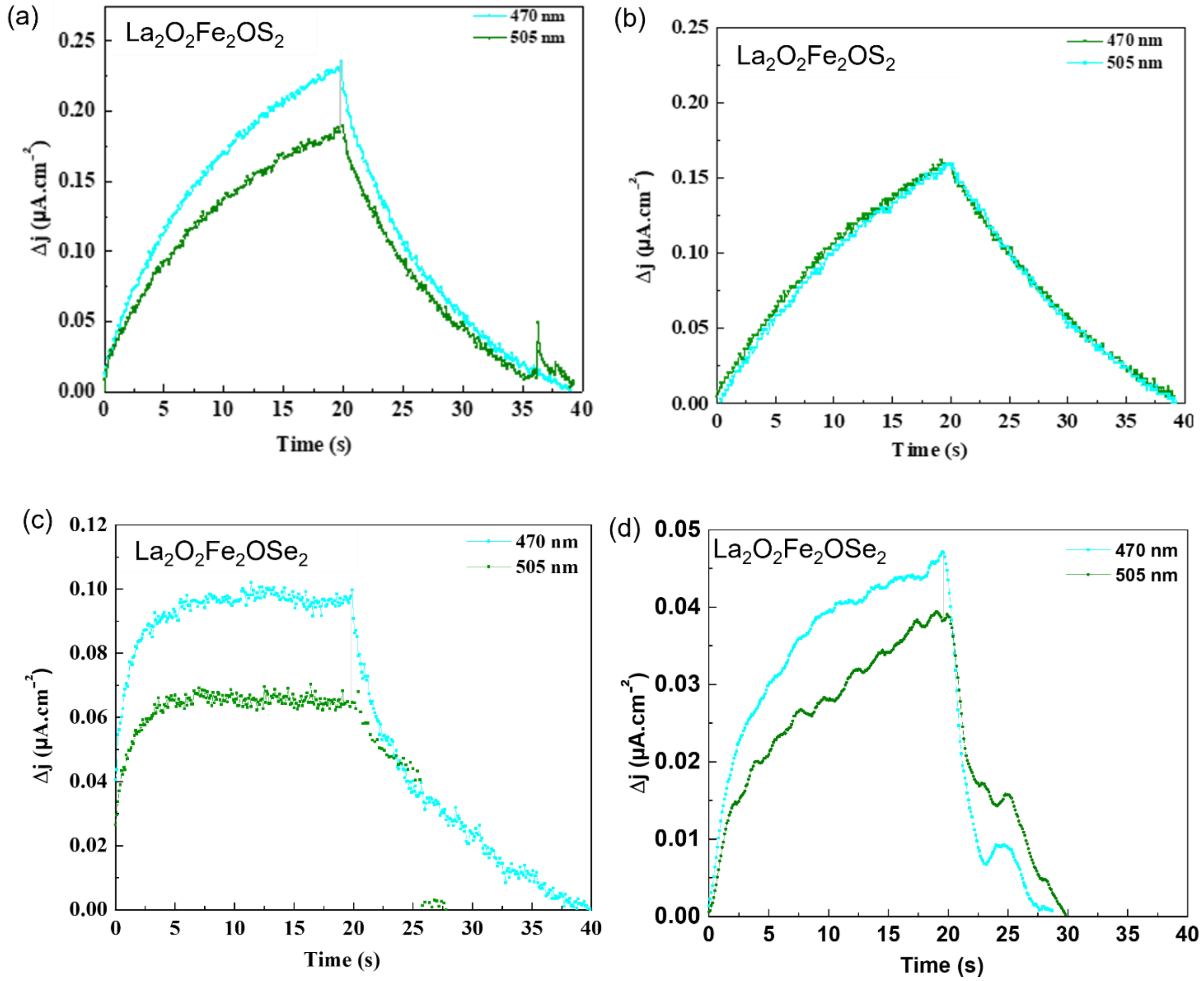

Figure SI6 Transient photocurrent response *vs.* wavelengths (constant light intensity ϕ_0=42 mW cm^{-2}) for $La_2O_2Fe_2OS_2$ with (a) $V_{bias} = 0.4$ V and (b) $V_{bias} = 0.0$ V; and for $La_2O_2Fe_2OSe_2$ with (c) $V_{bias} = 0.4$ V and (d) $V_{bias} = 0.0$ V.

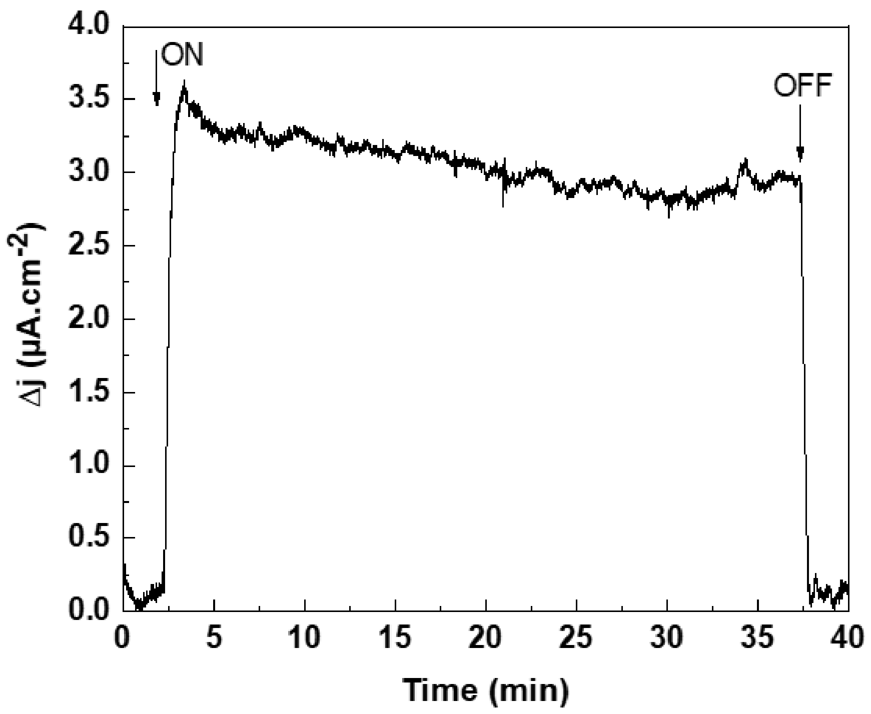

Figure SI7 Current density variation for $La_2O_2Fe_2OS_2$ at $V_{bias} = 0.6$ V under solar light excitation with 40 minutes exposure time.

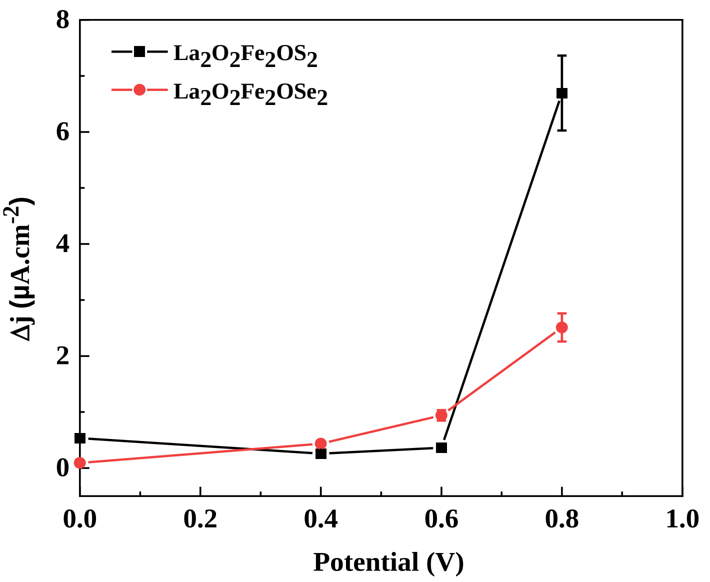

Figure SI8 Evolution of the transient photocurrent response *vs.* potential of $La_2O_2Fe_2OS_2$ and $La_2O_2Fe_2OSe_2$.

The photocurrent increased with the intensity of the luminous flux according to a classical power law behaviour[5] with $\Delta j = 8.63\times10^{-3}(2)\Phi^{0.60(2)}$ (Figure SI9).

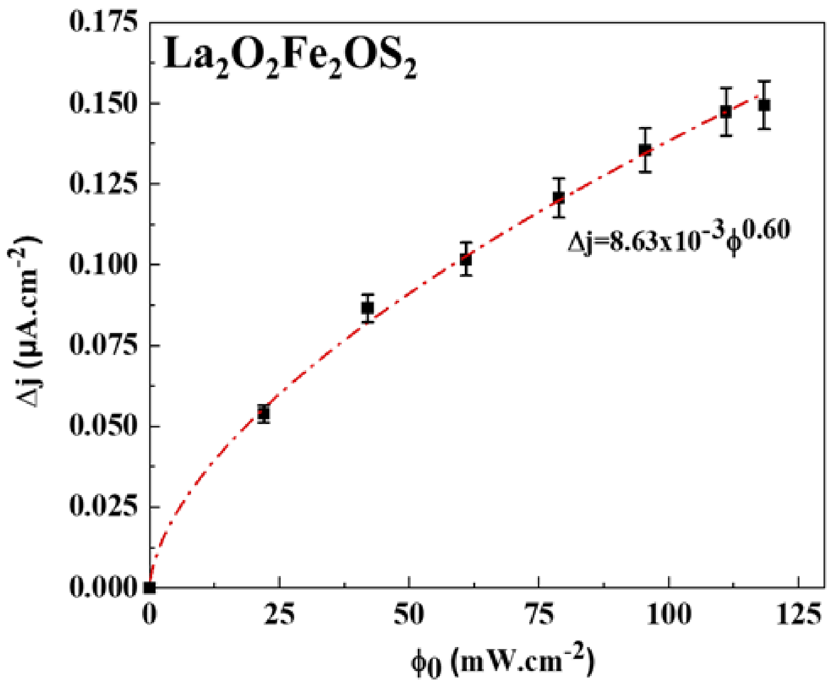

Figure SI9 Evolution of the photocurrent density with the power density of light (V_{bias}= 0 V under a 450 nm excitation) for $La_2O_2Fe_2OS_2$.

1. Butler, M. A.; Ginley, D. S., Prediction of Flatband Potentials at Semiconductor-Electrolyte Interfaces from Atomic Electronegativities. *Journal of The Electrochemical Society* **1978,** *125* (2), 228.
2. Xu, Y.; Schoonen, M. A. A., The absolute energy positions of conduction and valence bands of selected semiconducting minerals. *American Mineralogist* **2000,** *85* (3-4), 543-556.
3. Castelli, I. E.; Landis, D. D.; Thygesen, K. S.; Dahl, S.; Chorkendorff, I.; Jaramillo, T. F.; Jacobsen, K. W., New cubic perovskites for one- and two-photon water splitting using the computational materials repository. *Energy & Environmental Science* **2012,** *5* (10), 9034-9043.
4. Zhu, J.-X.; Yu, R.; Wang, H.; Zhao, L. L.; Jones, M. D.; Dai, J.; Abrahams, E.; Morosan, E.; Fang, M.; Si, Q., Band Narrowing and Mott Localization in Iron Oxychalcogenides La2O2Fe2O(S, Se)2. *Phys. Rev. Lett.* **2010,** *104* (21), 216405.
5. Shaikh, S. K.; Inamdar, S. I.; Ganbavle, V. V.; Rajpure, K. Y., Chemical bath deposited ZnO thin film based UV photoconductive detector. *Journal of Alloys and Compounds* **2016,** *664*, 242-249.
